# Anti-high mobility group box-1 (HMGB1) antibody attenuates delayed cerebral vasospasm and brain injury after subarachnoid hemorrhage in rats

**DOI:** 10.1038/srep37755

**Published:** 2016-11-24

**Authors:** Jun Haruma, Kiyoshi Teshigawara, Tomohito Hishikawa, Dengli Wang, Keyue Liu, Hidenori Wake, Shuji Mori, Hideo Kohka Takahashi, Kenji Sugiu, Isao Date, Masahiro Nishibori

**Affiliations:** 1 Department of Neurological Surgery, Okayama University Graduate School of Medicine, Dentistry and Pharmaceutical Sciences, Okayama, Japan; 2 Department of Pharmacology, Okayama University Graduate School of Medicine, Dentistry and Pharmaceutical Sciences, Okayama, Japan; 3 School of Pharmacy, Shujitsu University, Okayama, Japan; 4 Department of Pharmacology, Kinki University, Faculty of Medicine, Osaka-Sayama, Japan.

## Abstract

Although delayed cerebral vasospasm (DCV) following subarachnoid hemorrhage (SAH) is closely related to the progression of brain damage, little is known about the molecular mechanism underlying its development. High mobility group box-1 (HMGB1) plays an important role as an initial inflammatory mediator in SAH. In this study, an SAH rat model was employed to evaluate the effects of anti-HMGB1 monoclonal antibody (mAb) on DCV after SAH. A vasoconstriction of the basilar artery (BA) associated with a reduction of nuclear HMGB1 and its translocation in vascular smooth muscle cells were observed in SAH rats, and anti-HMGB1 mAb administration significantly suppressed these effects. Up-regulations of inflammation-related molecules and vasoconstriction-mediating receptors in the BA of SAH rats were inhibited by anti-HMGB1 mAb treatment. Anti-HMGB1 mAb attenuated the enhanced vasocontractile response to thrombin of the isolated BA from SAH rats and prevented activation of cerebrocortical microglia. Moreover, locomotor activity and weight loss recovery were also enhanced by anti-HMGB1 mAb administration. The vasocontractile response of the BA under SAH may be induced by events that are downstream of responses to HMGB1-induced inflammation and inhibited by anti-HMGB1 mAb. Anti-HMGB1 mAb treatment may provide a novel therapeutic strategy for DCV and early brain injury after SAH.

Delayed cerebral vasospasm (DCV) with subsequent cerebral ischemia, which usually develops 7 to 14 days after subarachnoid hemorrhage (SAH), is one of the major causes of morbidity and mortality in patients with ruptured cerebral aneurysms^1^,^2^. Despite numerous clinical and experimental studies, the pathophysiological mechanisms underlying DCV have not yet been elucidated.

Toll like receptors (TLRs), nuclear factor-kappa B (NF-κB), interleukin-1 beta (IL-1β) and tumor necrosis factor-alpha (TNF-α) have been shown to participate in the damage-induced inflammatory process after SAH, and to be involved in the breakdown of the blood brain barrier (BBB) in SAH^3^,^4^,^5^,^6^,^7^. Although inhibitors of these pro-inflammatory factors have been evaluated for their potential neuroprotective effects in experimental animal studies, the relationship between inflammation and cerebral vasospasm is not clearly understood^8^,^9^,^10^,^11^,^12^,^13^.

High mobility group box-1 (HMGB1), originally identified as a non-histone chromatin DNA-binding protein, is now recognized as a typical representative of the damage-associated molecular patterns (DAMPs)^14^,^15^. Once HMGB1 is released from different sources—such as necrotic cells or activated macrophages—into the extracellular milieu, the inflammatory responses are promoted through the stimulation of plural receptors such as TLR2/4 or receptor for advanced glycation endproducts (RAGE) on target cells^16^,^17^,^18^.

Recently, we found that the translocation and release of HMGB1 from neuronal nuclei to the extracellular space occurs both in ischemic and traumatic brain injuries, and that a neutralizing monoclonal antibody (mAb) against HMGB1 significantly ameliorates the resultant brain injuries by protecting against the BBB breakdown and reducing the inflammatory responses induced by the released HMGB1^19^,^20^,^21^.

Several studies have shown that plasma levels of HMGB1 were significantly elevated and remained at high levels during the post-SAH period^22^,^23^,^24^. These data strongly suggest that HMGB1 may play a crucial role in early brain injury and DCV after SAH.

In the present study, we demonstrate that treatment with anti-HMGB1 mAb dramatically attenuated the progression of DCV in a rat SAH model through the inhibition of HMGB1 translocation in arterial smooth muscle cells, suppression of up-regulation of vasocontractile receptors and diminution of vascular inflammatory responses, in association with the improvement of neurological symptoms. Therefore, treatment with anti-HMGB1 mAb may be an effective therapeutic strategy for SAH-induced vasospasm as well as ischemia-induced brain injury.

## Results

### Effects of Anti-HMGB1 mAb on Vasospasm after SAH

Cerebral vasospasm in SAH rats was evaluated by measuring the diameter of the basilar artery (BA) on computed tomographic angiography (CTA) images from the individual rats 48 hr before and after blood injection into the cisterna magna. Physiological parameters in the SAH rats are shown in Supplementary table S1. Anti-HMGB1 mAb or control IgG was administered to SAH rats at both 5 min and 24 hr after blood injection.

**Figure 1:**
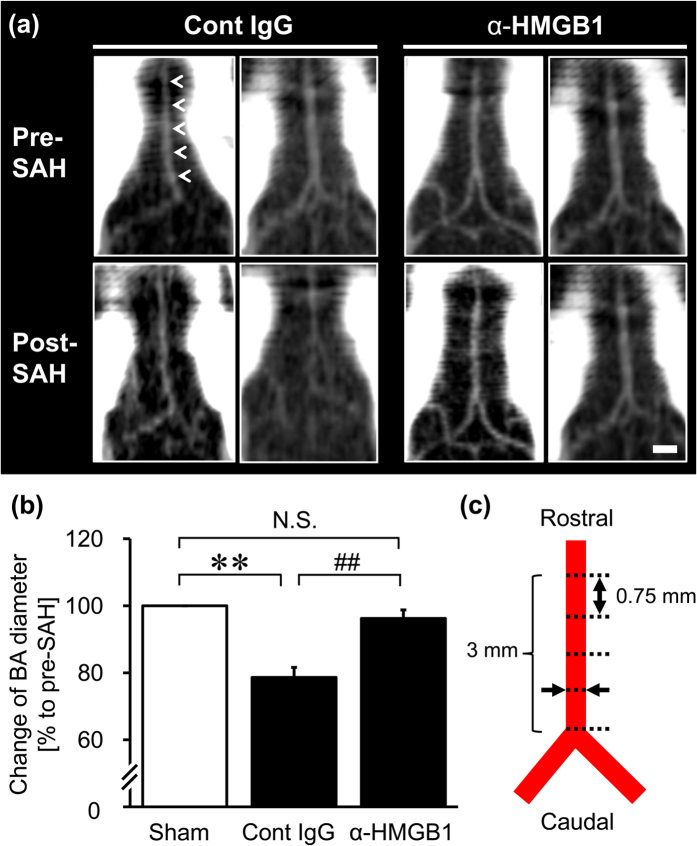
Effects of anti-HMGB1 mAb on the vasocontractile morphology of BA in SAH rats. CTA images of BA were observed at 48 hr before and after the SAH in rats administered anti-HMGB1 mAb or control IgG at both 5 min and 24 hr after blood injection. (**a**) Representative horizontal CTA images of the BA (arrowheads) from individual rats in the control IgG-treated group and the anti-HMGB1 mAb-treated group before SAH (upper panels) and after SAH (lower panels) are shown. (**b**) The magnitude of vasoconstriction was evaluated according to the ratio of the post-SAH to the pre-SAH BA diameter. (**c**) A schematic image of BA is shown. The diameter of BA in individual rats was measured at 5 points, as indicated by the dotted lines and the arrows. The scale bar indicates 1 mm. Results are shown for the sham group (Sham, n = 3), the control IgG-treated group (Cont IgG, n = 8), and the anti-HMGB1 mAb-treated group (α-HMGB1, n = 8). Values represent the means ± SE. **P < 0.01 compared with the sham group. ^##^*P* < 0.01 compared with the control IgG-treated group.

As shown in Fig. 1, 48 hr after the blood injection, a constant contraction of BA was observed in control IgG-treated rats. The average diameter of BA was reduced to 78.6 ± 3.0% of the pre-treatment values. The administration of anti-HMGB1 mAb (1 mg/kg, twice) significantly reversed the contractile response of BA to 96.2 ± 2.5% (Fig. 1a,b).

### Effects of Anti-HMGB1 mAb on Localization and Expression of HMGB1 in the BA of SAH Rats

**Figure 2:**
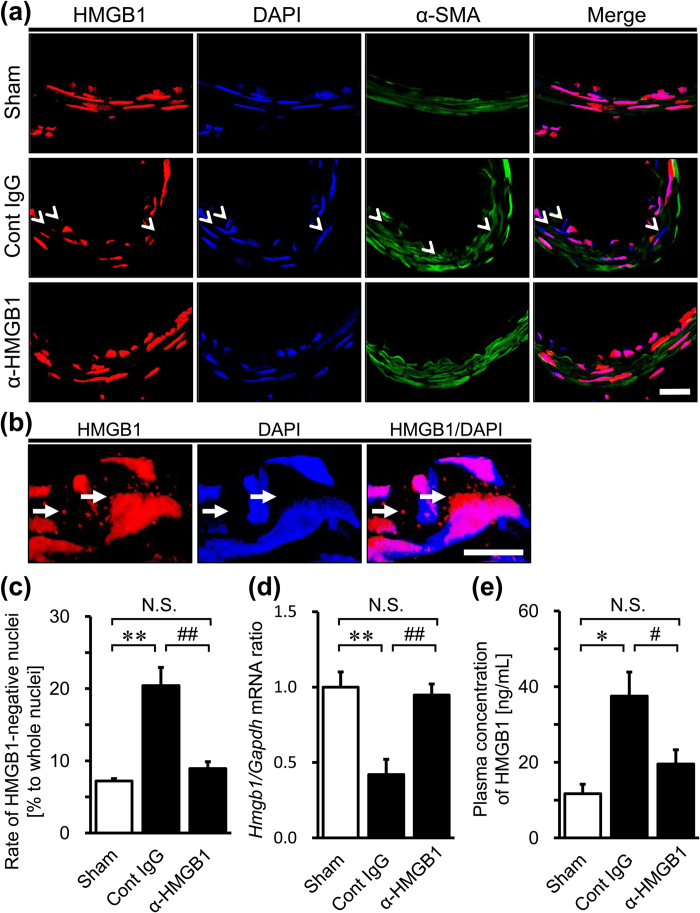
Effects of anti-HMGB1 mAb on mobilization of HMGB1 in SAH rats. Effects of anti-HMGB1 mAb were examined at 48 hr after the SAH in rats treated with anti-HMGB1 mAb or control IgG at both 5 min and 24 hr after blood injection. (**a**) The whole brain including the BA was prepared as coronal sections, and subjected to immunostaining for HMGB1 (Alexa Fluor 555) or α-smooth muscle actin (α-SMA, a marker protein of vascular smooth muscle cell) (Alexa Fluor 488) in the BA. All sections were counterstained with DAPI. Representative images of the BA in each group are shown. Arrowheads indicate the nuclei staining negative for HMGB1 in VSMCs. (**b**) Representative images of the HMGB1 translocation (arrows) to the cytoplasm from the nucleus in the control IgG-treated group are shown. (**c**) Nuclear localization of HMGB1 in VSMCs was evaluated by the ratio of the nuclei staining negative for HMGB1 to whole nuclei on immunofluorescent staining. (**d**) Total RNA was extracted from the isolated BA and subjected to quantitative RT-PCR for analysis of the expression of HMGB1 mRNA. GAPDH was used as a housekeeping gene. (**e**) The plasma concentration of HMGB1 was measured by ELISA. The scale bars indicate 20 μm. Results are shown for the sham group (Sham, n = 3, 4, 5 in (**a–e**), respectively), the control IgG-treated group (Cont IgG, n = 5, 4, 9 in (**a–**e), respectively), and the anti-HMGB1 mAb-treated group (α-HMGB1, n = 5, 4, 11 to in (**a–e**), respectively). Values represent the means ± SE. *P < 0.05, **P < 0.01 compared with the sham group. ^#^*P* < 0.05, ^##^*P* < 0.01 compared with the control IgG-treated group. N.S.: Not significant.

In the sham group, strong HMGB1 immunoreactivities were observed in the nuclei of vascular smooth muscle cells (VSMCs) of the BA (Fig. 2a upper panels). In the control IgG-treated group at 48 hr after SAH, some of the VSMCs were depleted of nuclear HMGB1. Generally, HMGB1 immunoreactivities in VSMCs of the BA were more faint than in the sham group and, unlike in the sham group, were present in the marginal zone of each nucleus (Fig. 2a middle panels). These results suggest the extracellular translocation of HMGB1 in VSMCs. We also observed cytoplasmic vesicle-like HMGB1 immunoreactivity in the VSMCs of the control IgG-treated group (Fig. 2b), which was considered to indicate the extranuclear translocation of HMGB1. On the other hand, the administration of anti-HMGB1 mAb restored most of the intranuclear localization of HMGB1 in VSMCs (Fig. 2a lower panels).

The reduction of nuclear HMGB1 protein observed immunohistochemically was confirmed quantitatively by counting the number of HMGB1-negative and DAPI-positive nuclei. A significantly higher number of HMGB1-negative nuclei was observed in the SAH control rats compared with the sham rats (Supplementary Figure S1). We did not find any difference in the distribution of HMGB1-negative nuclei along the rostro-caudal axis. The administration of anti-HMGB1 mAb suppressed the nuclear reduction of HMGB1 levels (Fig. 2c and Supplementary Figure S1). Quantitative RT-PCR for the expression of HMGB1 mRNA revealed that anti-HMGB1 mAb greatly suppressed the down-regulated expression of HMGB1 mRNA in the BA under SAH (Fig. 2d).

### Effects of Anti-HMGB1 mAb on the Plasma HMGB1 in SAH Rats

In the control IgG-treated group, the plasma level of HMGB1 was significantly higher than that in the sham group (37.5 ± 6.4 ng/mL versus 11.7 ± 2.5 ng/mL). On the other hand, the plasma level of HMGB1 in the anti-HMGB1 mAb-treated group (19.5 ± 3.8 ng/mL) was significantly lower than that in the control IgG-treated group (Fig. 2e).

### Effects of Anti-HMGB1 mAb on the Expression of Vasoconstriction- and Inflammation-Mediating Molecules in SAH Rats

**Figure 3:**
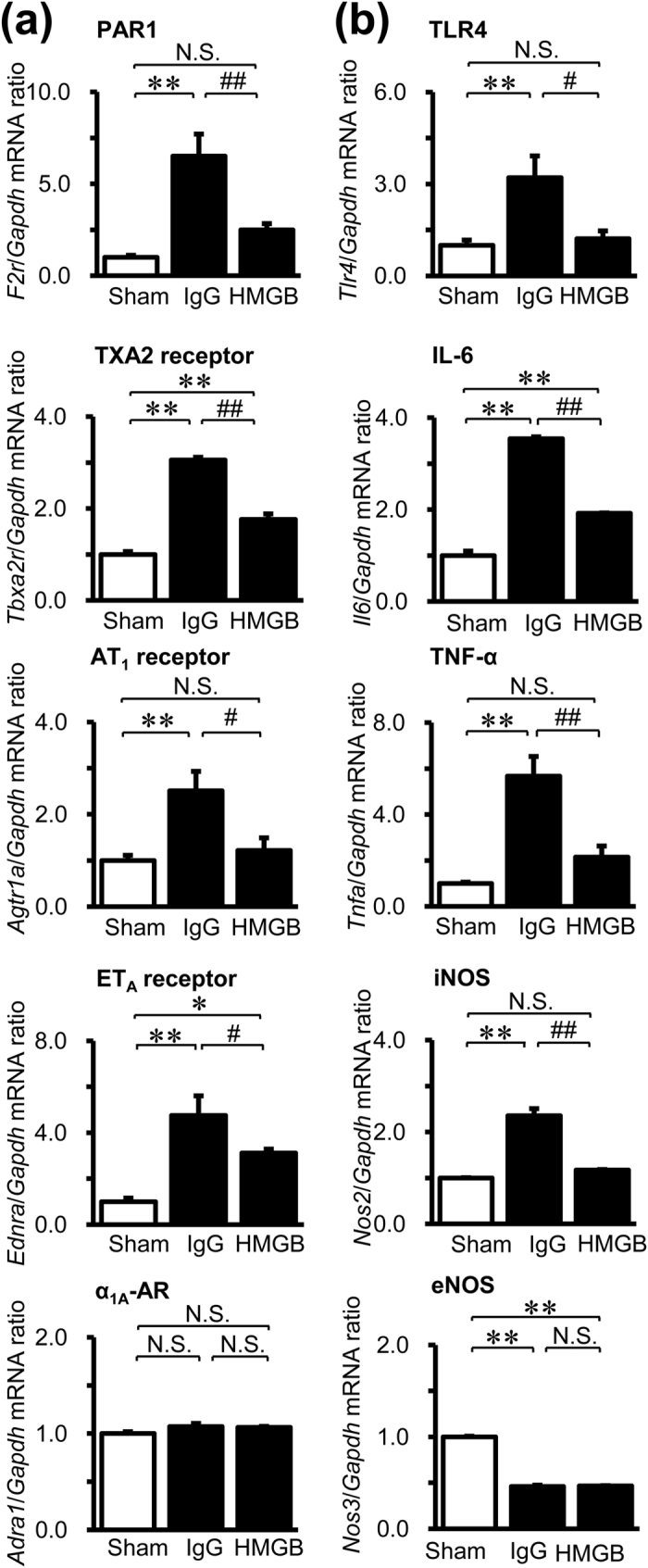
Effect of anti-HMGB1 mAb on gene expression in the BA of SAH rats. The BA was isolated at 48 hr after the SAH in rats treated with anti-HMGB1 mAb or control IgG at both 5 min and 24 hr after blood injection. Total RNA was extracted from the isolated BA and subjected to quantitative RT-PCR for analysis of the expression of vasoconstriction-mediating molecules (**a**) such as PAR1, TXA2 receptor, AT_1_ receptor, ET_A_ receptor, and α_1A_-AR, and inflammatory mediators (**b**) such as TLR4, IL-6, TNF-α, iNOS, and eNOS. GAPDH was used as a housekeeping gene. Results are shown for the sham group (Sham, n = 4), the control IgG-treated group (IgG, n = 4), and the anti-HMGB1 mAb-treated group (HMGB, n = 4). Values represent the means ± SE. *P < 0.05, **P < 0.01 compared with the sham group. ^#^*P* < 0.05, ^##^*P* < 0.01 compared with the control IgG-treated group. N.S.: Not significant.

To analyze the anti-vasocontractile mechanism underlying the effects of anti-HMGB1 mAb, we examined the expression of vasoconstriction- and inflammation-mediating molecules in the BA at 48 hr after SAH using quantitative RT-PCR (Fig. 3). In the control IgG-treated group, the gene expressions of protease-activated receptor-1 (PAR1, thrombin receptor), thromboxane A_2_ (TXA2) receptor, angiotensin II type 1 (AT_1_) receptor and endothelin type A (ET_A_) receptor, but not alpha-1A adrenergic receptor (α_1A_-AR), were up-regulated in the BA compared with those in the sham group (Fig. 3a). These up-regulations of gene expressions were significantly suppressed by the treatment with anti-HMGB1 mAb. Similarly, the increases in the expressions of toll-like receptor 4 (TLR4), interleukin-6 (IL-6), tumor necrosis factor-alpha (TNF-α) and inducible nitric oxide synthase (iNOS) after SAH were also significantly lower in the anti-HMGB1 mAb-treated group (Fig. 3b). On the other hand, the expression of endothelial nitric oxide synthase (eNOS) was significantly lower in both the control IgG- and the anti-HMGB1 mAb-treated group when compared with the sham group.

Consistent with the results of gene expression in Fig. 3, immunoblot analysis showed a similar alteration of protein content on PAR1 in the BA preparation (Supplementary Figure S2).

### Effects of Anti-HMGB1 mAb on the Contractile Response to Thrombin in the Isolated BA from SAH Rats

**Figure 4:**
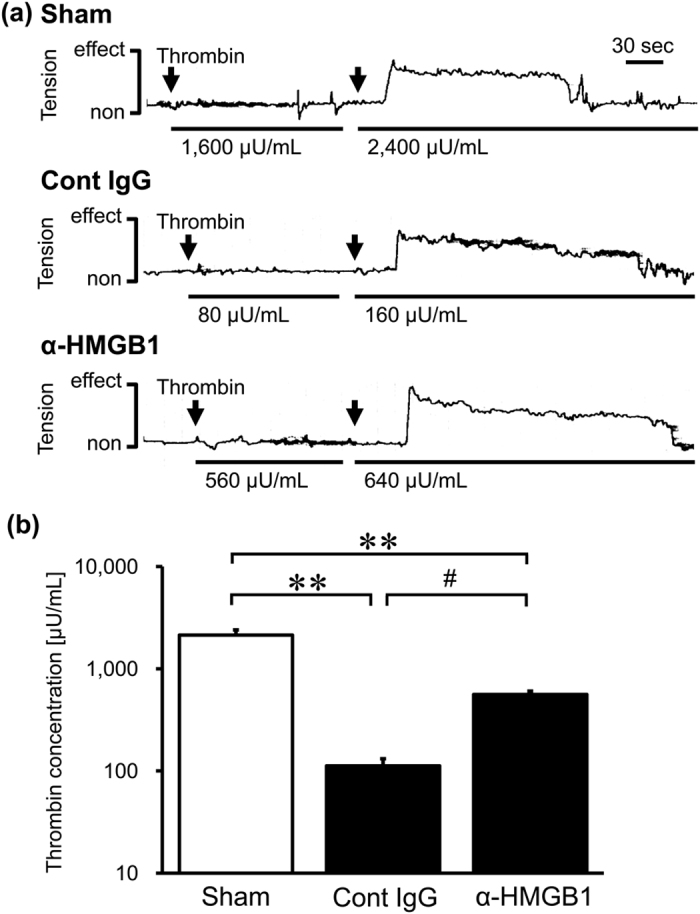
Effect of anti-HMGB1 mAb on the contractile response to thrombin on the isolated BA from SAH rats. The BA was isolated at 48 hr after the SAH in rats administered anti-HMGB1 mAb or control IgG at both 5 min and 24 hr after blood injection. (**a**) Tension responses to thrombin stimulation in a BA segment are shown as representative recordings. Arrows show the time point of accumulative thrombin addition. Solid lines under the recording waveform indicate the stimulating phase at the indicated concentration of thrombin. (**b**) The minimum concentration of thrombin stimuli required for the initial contractile response was evaluated. Results are shown for the sham group (Sham, n = 3), the control IgG-treated group (Cont IgG, n = 5), and the anti-HMGB1 mAb-treated group (α-HMGB1, n = 5). Values represent the means ± SE. **P < 0.01 compared with the sham group. ^#^*P* < 0.05 compared with the control IgG-treated group.

The vasocontractile response to thrombin was examined *ex vivo* using the isolated BA from SAH rats to assess changes in the vasocontractile property after SAH and their prevention by anti-HMGB1 mAb administration. Under a stepwise increase in thrombin concentrations, the thresholds of thrombin concentrations were determined on each preparation from three groups (Fig. 4). The isolated BA from the sham group did not show the contractile response to thrombin until the concentration reached 2133 ± 267 μU/mL and above. In the control IgG-treated group, the contractile response of the isolated BA occurred at much lower concentrations of 112 ± 20 μU/mL thrombin, demonstrating the hyper-contractile property. On the other hand, the threshold of the thrombin concentration in the isolated BA from the anti-HMGB1 mAb-treated group was significantly higher than that in the control IgG-treated group (560 ± 44 μU/mL).

### Effects of Anti-HMGB1 mAb on Activated Microglia in the Brain of SAH Rats

**Figure 5:**
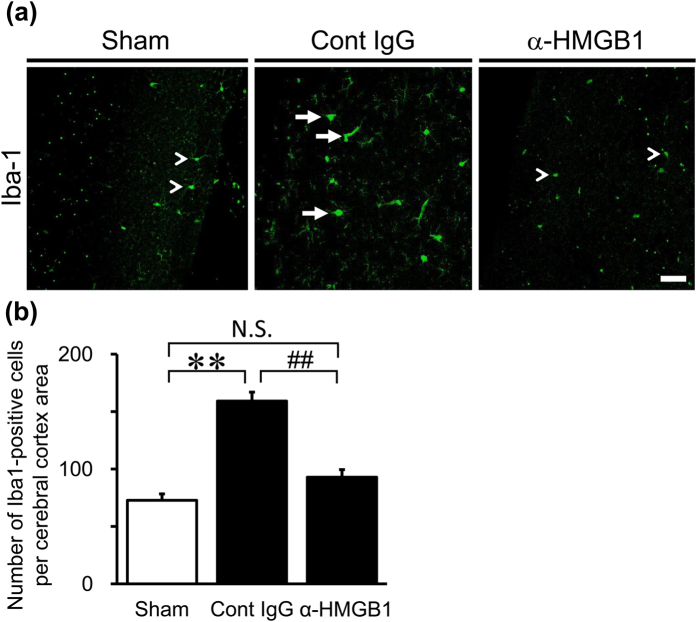
Effect of anti-HMGB1 mAb on microglial activity in the brain of SAH rats. Coronal sections of the whole brain were prepared at 48 hr after the SAH in rats treated by administration of anti-HMGB1 mAb or control IgG at both 5 min and 24 hr after blood injection. (**a**) Representative immunostaining images for Iba-1 (Alexa Fluor 488) are shown in the cerebral cortex area. Arrowheads and arrows in (**a**) indicate ramified and ameboid morphology of Iba1-positive microglia, respectively. (**b**) The proliferation of microglia was quantified by the number of Iba1-positive microglia existing around the whole surface area of the cerebral cortex. The scale bar indicates 50 μm. Results are shown for the sham group (Sham, n = 3), the control IgG-treated group (Cont IgG, n = 5), and the anti-HMGB1 mAb-treated group (α-HMGB1, n = 5). Values represent the means ± SE. **P < 0.01 compared with the sham group. ^##^*P* < 0.01 compared with the control IgG-treated group. N.S.: Not significant.

To confirm the inflammatory conditions in a wide region of the central nervous system (CNS) under SAH, immunohistochemical staining of the cerebral cortex was performed. Iba1-positive microglia in the sham group almost existed as the ramified morphological phenotype—namely, as resting microglia. A significantly greater number of microglia in the control IgG-treated group were observed as compared with the sham group. In particular, most of the Iba1-positive microglia in the control IgG-treated group were activated microglia based on the ameboid morphology. On the other hand, the number and morphological activity of microglia in the anti-HMGB1-treated group clearly recovered to the levels observed in the sham group (Fig. 5).

Numerous TUNEL-positive cells were also observed in the brains of the control IgG-treated group but not in the sham group, and these cells were clearly reduced by the administration of anti-HMGB1 mAb (Supplementary Figure S3).

### Effects of Anti-HMGB1 mAb on Neurological Symptoms and Body Weight after SAH

**Figure 6:**
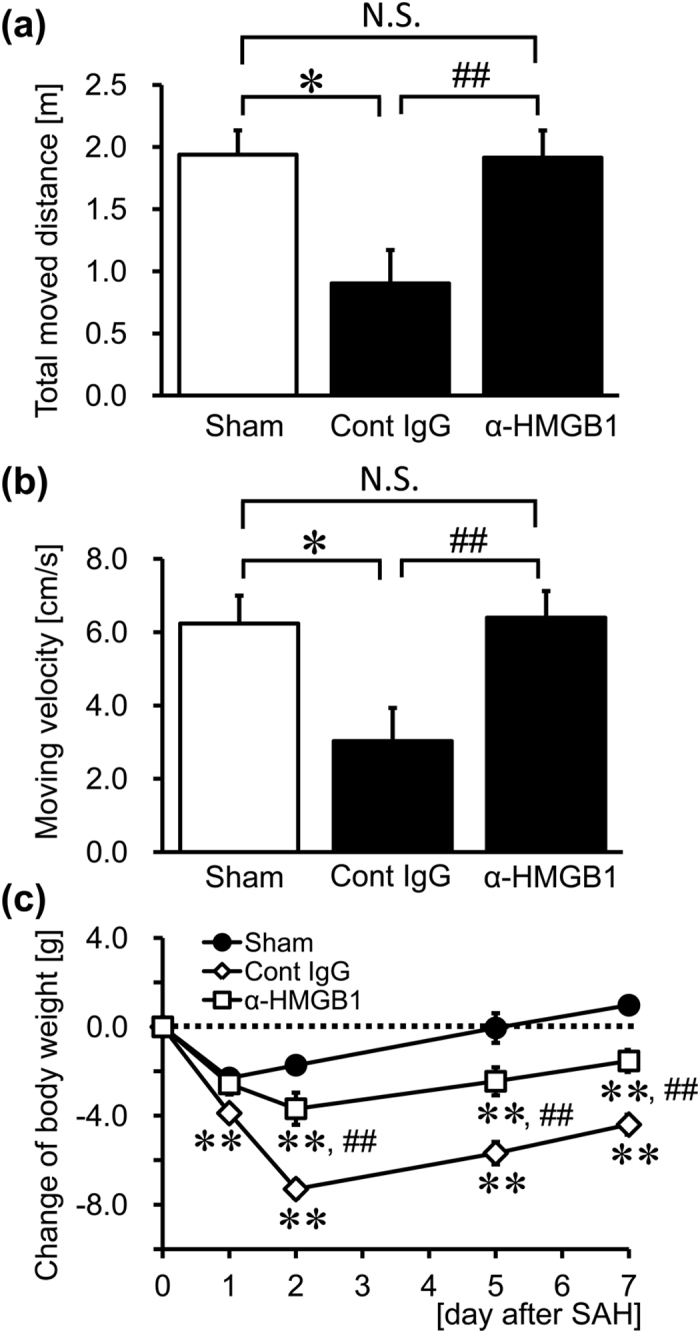
Effect of anti-HMGB1 mAb on neurological symptoms and body weight after SAH. An open field test was performed at 48 hr after the SAH in rats administered anti-HMGB1 mAb or control IgG at both 5 min and 24 hr after blood injection. The total distance moved (**a**) and moving velocity (**b**) were measured to assess locomotor activity. (**c**) Changes in the body weights of rats were also measured once daily over the 7 days after the SAH procedure. Results are shown for the sham group (Sham, n = 4), the control IgG-treated group (Cont IgG, n = 6), and the anti-HMGB1 mAb-treated group (α-HMGB1, n = 6). Values represent the means ± SE. *P < 0.05, **P < 0.01 compared with the sham group. ^##^*P* < 0.01 compared with the control IgG-treated group. N.S.: Not significant.

Impairments of coordinated locomotor activity after SAH were assessed by using an open field test in which the rats were exposed to a new environment. Both the total distance moved over 5 min and the average moving velocity were lower in the control IgG-treated group than in the sham group. The lower locomotor activities in SAH control rats were restored to healthy levels by the administration of anti-HMGB1 mAb (Fig. 6a,b).

We next measured the changes in body weight in the experimental rats over the first week after SAH to evaluate the general condition of rats (Fig. 6c). The extent of weight loss in the control IgG-treated group was significantly greater than in the sham group at 1 to 7 days after SAH. Although the body weights in the anti-HMGB1 mAb-treated group were lower than those in the sham control group at 2 to 7 days after SAH, the extent of weight loss was significantly smaller than in the control IgG-treated group at 2 to 7 days after SAH.

### Evaluation of the Effects of Anti-HMGB1 mAb Treatment in Relation to the Therapeutic Time Window

Finally, we examined the effects of anti-HMGB1 mAb treatment starting at 3 hr after the onset of SAH. Anti-HMGB1 mAb or control IgG was administered to SAH rats at both 3 and 27 hr after blood injection.

In the control IgG-treated rats, CTA images of BA showed a similar BA vasospasm 48 hr after the blood injection, as observed in Fig. 1a,b. The average diameter of BA was reduced to 79.4 ± 1.3% of the pre-treatment values. The administration of anti-HMGB1 mAb significantly reversed the contractile response of BA, resulting in a BA diameter that was 86.4 ± 0.6% of the pre-treatment value.

**Figure 7:**
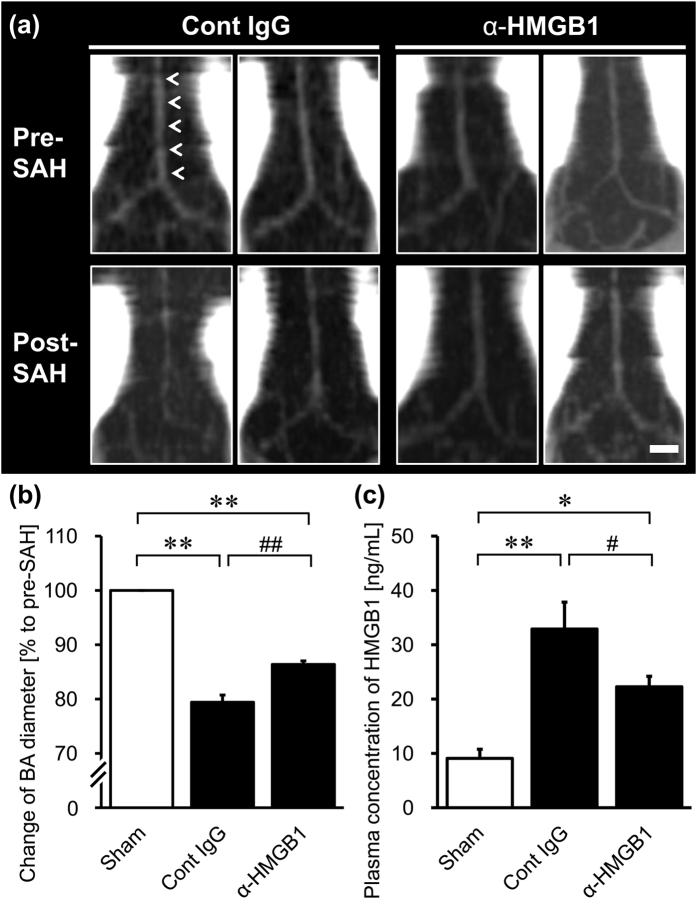
Evaluation of the effects of anti-HMGB1 mAb treatment with respect to the therapeutic time window. Rats were administered anti-HMGB1 mAb or control IgG at both 3 hr and 27 hr after blood injection. (**a**) CTA images of the BA were observed at 48 hr before and after the SAH. Representative horizontal CTA images of the BA (arrowheads) from individual rats in the control IgG-treated group and the anti-HMGB1 mAb-treated group before SAH (upper panels) and after SAH (lower panels) are shown. (**b**) The magnitude of vasoconstriction was evaluated according to the ratio of the post-SAH to the pre-SAH BA diameter. (**c**) The plasma concentrations of HMGB1 were measured by ELISA. The scale bar indicates 1 mm. Values represent the means ± SE. **P < 0.01 compared with the sham group. ^#^*P* < 0.05, ^##^*P* < 0.01 compared with the control IgG-treated group. For the CTA imaging, the sham-, control IgG- and anti-HMGB1 groups included 3, 5 and 5 rats, respectively. For the plasma HMGB1 determination, the sham-, control IgG- and anti-HMGB1 groups included 6, 8 and 9 rats, respectively.

The increase of plasma HMGB1 level in the control IgG-treated group (32.9 ± 4.9 ng/mL versus 9.1 ± 1.7 ng/mL) was also significantly reduced by the treatment with anti-HMGB1 mAb (22.3 ± 1.9 ng/mL) (Fig. 7c).

These results suggest that anti-HMGB1 mAb treatment may have a therapeutic time window of at least 3 hr after brain injury.

## Discussion

In the present study, CTA imaging of live rats pre- and post-SAH clearly showed that treatment with anti-HMGB1 mAb significantly suppressed the delayed vasoconstriction 2 days after SAH in rats (Figs 1 and 7). In human patients, early brain injury and delayed vasospasm are the main causes of mortality and morbidity after SAH, even when the endovascular coil embolism or the surgical clipping of aneurysm is successful^25^,^26^. In such cases, the extent and severity of the ischemia-induced brain injury determine the neurological deficits and overall poor outcome^27^.

Therefore, the development of therapies for early brain injury and delayed vasospasm after SAH has been one of the main goals in SAH treatment for decades. The smooth muscle relaxants, such as calcium antagonists, Rho-kinase inhibitors and endothelin receptor antagonists, have been used clinically for the treatment of vasospasm, but improvements in the overall outcome have not yet been achieved^28^,^29^,^30^. One of the reasons for the lack of clear effects of the smooth muscle relaxants in clinical practice may be the complicated pathogenesis of brain injury in SAH. For example, it has been reported that inflammatory responses were closely associated with brain injury and ischemia induced by vasospasm in SAH patients and experimental animals^31^. The induction of inflammatory cytokines, ROS-triggered inflammatory events and disruption of the blood brain barrier under SAH all contribute to brain inflammation, leading to hypoperfusion and hypoxia in the affected areas. However, the causal relationship between subarachnoid hemorrhage, brain inflammatory responses and delayed vasospasm remains poorly understood^32^.

There is a large body of clinical data showing increases in HMGB1 in the CSF and blood of SAH patients, and these changes have been closely correlated with the severity of neurological symptoms and long-term outcome of patients^22^,^33^. These clinical findings strongly suggest that HMGB1 is involved in the pathogenesis of SAH-induced brain injury.

In the present study, the administration of anti-HMGB1 mAb to the SAH rats inhibited the translocation and release of HMGB1 in smooth muscle cells in addition to reducing HMGB1 levels in the BA. The concomitant determination of plasma HMGB1 also indicated that the anti-HMGB1 mAb significantly inhibited the increase in plasma HMGB1 levels in the SAH rats (Figs 2 and 7). These results suggest that the bleeding into the subarachnoid space triggered HMGB1 release from VSMCs in the affected arterial walls, and that one of the origins of the elevated plasma HMGB1 may be smooth muscle cells in the affected arterial walls. The blockade by anti-HMGB1 mAb of HMGB1 translocation/release in arterial walls, the elevation of plasma HMGB1 and the delayed vasospasm implied not only a causal relationship between these events but also the presence of a vicious cycle of HMGB1-induced HMGB1 release as suggested in ischemic and traumatic brain injuries^19^,^20^. That is, in the previous studies, it was suggested that the initial release of HMGB1 facilitated further release of HMGB1 from neuronal nuclei^19^,^20^. A similar cyclic event could take place in VSMCs as well. Another possibility is that an increase in plasma HMGB1 might be involved in establishing the vicious cycle, since anti-HMGB1 treatment has been shown to efficiently reduce the plasma levels of HMGB1 in all cases of brain ischemia^19^, brain trauma^20^ and SAH.

**Figure 8:**
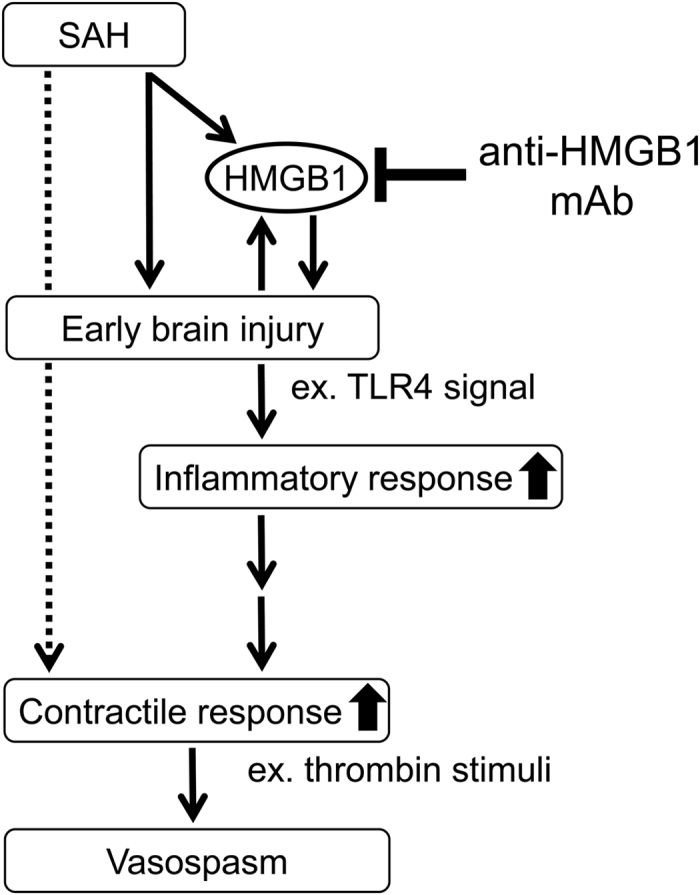
Schema of the effects of anti-HMGB1 mAb on delayed cerebral vasospasm (DCV) and subarachnoid hemorrhage (SAH). In the early stage after SAH, administration of anti-HMGB1 mAb suppresses a vicious cycle for extracellular HMGB1-induced facilitation of HMGB1 release from cell nuclei on hemorrhage-damaged regions including the cerebral cortex and BA, inhibiting downstream activity in the HMGB1-induced TLR4 pathway. Subsequently, anti-HMGB1 mAb also suppresses the enhancement of vasocontractile response induced by the up-regulation of inflammatory mediators. As a result, the onset of DCV may be prevented along with the reduction of the cerebral inflammation.

The HMGB1 released from smooth muscle cells may diffuse to the surrounding cells and induce plural events through two receptors in the vascular walls: RAGE and TLR2/4. One group of events would consist of increases in the expression of a series of inflammation-related molecules, while the other would consist of up-regulations of vasocontraction-inducing receptors, based on the finding that anti-HMGB1 mAb treatment significantly diminished both types of responses (Fig. 3). Thus, these findings suggest that HMGB1 release is present upstream of these two branches of events, and that HMGB1 in early brain injury acts as a primary initiation factor to accelerate the expansion of various pathogeneses, linking SAH to the subsequent induction of inflammation-related molecules and vasoconstriction-inducing receptors. Therefore, it could be concluded that HMGB1 is an excellent therapeutic target for the prevention of inflammation-induced vasospasm and improvement of contractile conditions (Fig. 8). In fact, we found that anti-HMGB1 mAb treatment was effective within a therapeutic time window of 3 hr in this animal model (Fig. 7), implying its possible clinical use.

Receptor signaling mediated by both TLR2/4 and RAGE activates plural adaptor molecules, such as myeloid differentiation primary response protein 88 (MyD88), TIR-domain containing adaptor protein (IRAK), RAS-related C3 botulinus toxin substrate 1 (Rac1) and cell division cycle 42 (CDC42), whereas their down-stream signals may cause the activation of NF-κB in common, leading to the induction of inflammation-related molecules, such as adhesion molecules (e.g., intercellular adhesion molecule-1 (ICAM-1), vascular cell adhesion molecule-1 (VCAM-1)) and cytokines (e.g., TNF-α, IL-1β and IL-6)^18^. Several reports have independently demonstrated that TLR2/4 and RAGE were involved in the inflammatory responses after SAH^3^,^34^,^35^,^36^,^37^. Therefore, it is likely that both TLR2/4 and RAGE mediate the action of HMGB1. Further studies will be needed to determine the relative importance of the receptors involved.

The *ex vivo* experiments using the isolated BA clearly showed that anti-HMGB1 mAb treatment significantly reversed the thrombin threshold in contractile response through PAR1 activation in the SAH rats (Fig. 4), strongly indicating that the normalization of the thrombin-induced contraction of the BA was due to a reduction of PAR1 expression and the relevant hyper-responsive state of smooth muscle cells. Kai, Maeda and their collaborators demonstrated that the isolated BA from SAH rabbits exhibited a higher contractile response to thrombin mediated by PAR-1 receptor stimulation. They attributed this augmented response to disorder of the calcium sequestration system and the resultant sensitization of contractile machinery^38^,^39^. They also observed that up-regulation of PAR-1 mRNA and PAR-1 protein were greater in the BA in SAH rabbits^38^, which was consistent with the present results in rats.

In addition to up-regulation of PAR1, we demonstrated the up-regulation of mRNAs for vasocontraction-inducing receptors such as TXA2 receptor, AT_1_ receptor and ET_A_ receptor in the BA of SAH rats (Fig. 3a). The concurrent changes in the expression of a diverse range of vasocontraction-inducing receptors and their sensitivity may be an important factor in vasospasm after SAH in patients as well as experimental animals, because the blocking of a single receptor has never shown clear beneficial effects in SAH patients^40^.

The marked inductions of inflammation-related genes in the BA of SAH rats, such as IL-6, TNF-α, TLR-4 and iNOS, were almost completely inhibited by anti-HMGB1 mAb (Fig. 3b). It has been speculated that the conditions induced by inflammation may be closely associated with facilitation of the contractile phenotype of smooth muscle cells. Therefore, it is likely that the strong suppression of inflammation-related events in the arterial walls by anti-HMGB1 mAb treatment contributes to the relaxation of arteries indirectly by inhibiting the phenotypic change of smooth muscle cells^41^. Further studies will be needed to clarify the linkage between the induction of inflammatory responses and the acquisition of the contractile phenotype in smooth muscle cells.

On the other hand, anti-HMGB1 mAb did not show any effects on the SAH-induced down-regulation of eNOS expression. Munakata and their collaborators demonstrated that the down-regulated expression of the eNOS gene in the BA of SAH rabbits was almost completely recovered by using the cyclooxygenase-2 (COX-2) selective inhibitor, suggesting the involvement of arachidonic acid metabolites in delayed vasospasm after SAH^42^. Thus, reduction of NO production by eNOS after SAH appeared to be mediated by COX-2 metabolites but not HMGB1. The combination therapy of anti-HMGB1 and COX-2 inhibition may produce additive effects on vasoconstriction after SAH.

The improvement of locomotor activity and weight loss recovery by anti-HMGB1 mAb in rats after SAH clearly reflected the amelioration of the general condition of rats (Fig. 6), and was consistent with the findings that anti-HMGB1 mAb treatment inhibited the apoptosis of neurons and the activation of microglia and astrocytes across a wide region of the cerebral cortex and brain stem^19^,^20^,^21^.

In conclusion, this study was the first to show the dynamic mobilization of HMGB1 from smooth muscle cells in the BA of SAH model rats. Anti-HMGB1 mAb therapy strongly inhibited the cascade of events, including the HMGB1 translocation/mobilization, up-regulation of vasocontraction-inducing receptors, and expression of inflammation-related molecules. Collectively, these effects of anti-HMGB1 mAb appeared to contribute to the relaxation of vasospasm under SAH. Anti-HMGB1 therapy would interrupt this cascade by neutralizing the extracellular HMGB1, thereby preventing inflammatory responses and strong vasospasm in the BA, and ultimately reducing the ischemia-induced brain damage and improving the neurological symptoms. The SAH-induced cascade of events may be effectively disrupted by anti-HMGB1 mAb therapy.

## Materials and Methods

### Animals and Surgical Procedures

All animal experiments were approved by the Institutional Animal Care and Use Committee of Okayama University, and performed in accordance with the guidelines of Okayama University on animal experiments. Adult male Wistar rats at 10 to 12 weeks old and weighing 300 to 350 g were used for the experiments. The procedures used for single-hemorrhage in the SAH rat model have been described previously^43^. After the SAH, the rats were intravenously administered an anti-HMGB1 mAb (#10-22, rat IgG2a subclass, 1 mg/kg) or class-matched control IgG (anti-keyhole limpet hemocyanin mAb) twice with a 24 hr interval^7^,^8^. To examine the therapeutic time window, the treatments were started 5 min and 3 hr after blood injection, respectively. Following treatment, at 48 hr after blood injection, the rats were sacrificed to obtain the whole brain, isolated basilar artery (BA) and plasma samples (see the schematic outline in Supplementary Figure S4).

### Computed Tomographic Angiography

Computed tomographic angiography (CTA) was performed with an X-ray CT system (Latheta LCT-200; Hitachi Aloka Medical, Tokyo, Japan) to measure the diameter of BA at 48 hr before and after SAH as described previously^44^,^45^,^46^. Processing and analysis of the CTA DICOM (digital imaging and communications in medicine)-formatted data were performed by using OsiriX software (open source). The diameter of BA was evaluated as the average value of 5 points at an interval of 0.75 mm in individual rats (Fig. 1c), using a double-blind procedure to prevent experimental bias.

### Enzyme-linked Immunosorbent Assay

A sensitive and specific anti-HMGB1 monoclonal antibody-based sandwich enzyme-linked immunosorbent assay (ELISA) was established. The capture and detection antibodies were produced by our group as described previously^21^.

### Immunohistochemistry

Immunohistochemical staining was performed as described previously^19^. A series of coronal sections were cut at a site 7 mm posterior from the bregma in the paraffin-embedded whole brain, including the BA. The sections were incubated with anti-HMGB1 mouse monoclonal antibody (R&D Systems, Minneapolis, MN, USA), anti-alpha smooth muscle actin (α**-**SMA) rabbit polyclonal antibody (Abcam, Cambridge, UK) or anti-Iba1 rabbit polyclonal antibody (Wako, Osaka, Japan). The sections were also counterstained with 4′,6-diamidino-2-phenylindole (DAPI). To analyze the proportion of HMGB1-negative nuclei in the BA, the numbers of HMGB1-negative nuclei and DAPI-positive nuclei were counted in a whole coronal section of BA from each rat (see the description in Supplementary Figure S1).

### Quantitative Reverse Transcription-Polymerase Chain Reaction

Total RNA was extracted from the isolated BA. Quantitative reverse transcription-polymerase chain reaction (quantitative RT-PCR) was performed as described previously^20^,^47^. The expression of GAPDH was used to normalize the amount of cDNA. The specificity of the PCR amplification product was confirmed by analyzing a melting curve. The gene-specific primers are described in Supplementary Table S2.

### Tension Measurement in the BA

The BA was isolated from the brain at 48 hr after SAH, and a ring preparation was made to examine the vasocontractile response to thrombin stimuli as described previously^10^,^38^. After the isolated BA was connected to a force transducer (U Gauge; Nihon Kohden, Tokyo, Japan) in an organ bath for continuous recording of isometric tension, 0.02 U/mL thrombin solution in a volume of 20 μL was added cumulatively over an interval of 3 min until the first contractile response was observed in the recording waveform. The cumulative concentration of thrombin required to induce the initial contractile response was calculated.

### Assessment of Physical Activity

The standard open field test was performed in an acrylic box (W120 cm × D120 cm × H60 cm) to assess the locomotor activity of rats at 48 hr after SAH. Rats were individually placed in the open field and allowed to locomote freely for 5 min. The behavior of rats was monitored by a CCD camera and analyzed by Ethovision XT software (Noldus, Wageningen, Netherlands) as a video tracking system. The body weights of rats were also measured once daily over the 7 days after SAH.

### Statistical Analysis

Statistical comparisons were performed using one-way ANOVA or repeated measures two-way ANOVA followed by the *post hoc* Bonferroni test with Ekuseru-Toukei 2010 (Social Survey Research Information, Tokyo, Japan), except that the quantitative RT-PCR (Fig. 3), locomotor activity (Fig. 6a,b) and plasma HMGB1 level (Fig. 7c) were analyzed using one-way ANOVA followed by the *post hoc* Fisher’s LSD test. The mean values of data are shown along with the standard error. *P* values less than 0.05 were considered statistically significant.

## Additional Information

**How to cite this article**: Haruma, J. *et al*. Anti-high mobility group box-1 (HMGB1) antibody attenuates delayed cerebral vasospasm and brain injury after subarachnoid hemorrhage in rats. *Sci. Rep.* **6**, 37755; doi: 10.1038/srep37755 (2016).

**Publisher’s note:** Springer Nature remains neutral with regard to jurisdictional claims in published maps and institutional affiliations.

## Supplementary Material

Supplementary Information
