# Supplementary material for: Anti-high mobility group box-1 (HMGB1) antibody attenuates delayed cerebral vasospasm and brain injury after subarachnoid hemorrhage in rats
Source: Sci Rep. 2016 Nov 24;6:37755. doi: 10.1038/srep37755 (PMC5121891; doi:10.1038/srep37755)
Supplement: Supplementary Information [file srep37755-s1.pdf]

ONLINE SUPPLEMENT

Supplementary Tables and Figures

Anti-high mobility group box-1 (HMGB1) antibody attenuates delayed cerebral vasospasm and brain injury after subarachnoid hemorrhage in rats.

Jun Haruma[1,2], MD; Kiyoshi Teshigawara[2], PhD; Tomohito Hishikawa[1], MD, PhD; Dengli Wang[2]; Keyue Liu[2], MD, PhD; Hidenori Wake[2], PhD; Shuji Mori[3], PhD; Hideo Kohka Takahashi[4], MD, PhD; Kenji Sugiu[1], MD, PhD; Isao Date[1], MD, PhD; Masahiro Nishibori[2], MD, PhD

[1] Department of Neurological Surgery, Okayama University Graduate School of Medicine, Dentistry and Pharmaceutical Sciences.

[2] Department of Pharmacology, Okayama University Graduate School of Medicine, Dentistry and Pharmaceutical Sciences.

[3] School of Pharmacy, Shujitsu University.

[4] Department of Pharmacology, Kinki University, Faculty of Medicine.

Correspondence to

Masahiro Nishibori, MD, PhD.

Department of Pharmacology, Okayama University Graduate School of Medicine,

Dentistry and Pharmaceutical Sciences, 2-5-1 Shikata-cho, Kita-ku, Okayama 700-8558, Japan.

Tel/FAX: +81-86-235-7140.

E-mail: mbori@md.okayama-u.ac.jp

Supplementary table S1: Physiological parameters in experimental SAH rats.

		Sham	Cont IgG	α-HMGB1
Pre - SAH	BP	92.0 ± 5.2	91.3 ± 5.2	90.7 ±4.9
	HR	302.8 ± 23.3	305.7 ± 14.1	306.0 ± 8.3
	ICP	N.D.	5.8 ± 0.6	5.1 ± 1.2
Post - SAH	BP	95.6 ± 11.4	109.8 ± 12.1	103.1 ± 11.6
	HR	311.2 ± 20.2	289.8 ± 17.1	305.2 ± 6.1
	ICP	N.D.	53.1 ± 6.1	68.5 ± 6.1
Blood gas sample	pH	7.5 ± 0.02	7.4 ± 0.02	7.5 ± 0.02
	pCO_2	41.0 ± 2.1	38.5 ± 2.5	34.0 ± 1.7
	pO_2	97.7 ± 0.6	98.7 ± 0.3	98.8 ± 0.3
	BE	4.2 ± 0.8	1.4 ± 0.7	1.0 ± 1.0
	HCO_3	28.4 ± 0.7	25.7 ± 0.9	24.6 ± 1.0*
	Na	137.4 ± 1.7	137.8 ± 0.7	140.8 ± 0.6
	K	4.0 ± 0.4	4.2 ± 0.1	4.0 ± 0.2
	iCa	1.3 ± 0.02	1.3 ± 0.02	1.3 ± 0.02
	Glu	233.4 ± 17.2	188.2 ± 29.6	196.6 ± 21.7
	Hct	40.4 ± 1.5	38.4 ± 0.5	39.0 ± 0.8
	Hb	12.9 ± 0.1	13.1 ± 0.2	13.3 ± 0.3

Blood pressure, heart rate, intracranial pressure were measured just before and after blood injection in SAH procedure. Blood gases were measured just the once after the blood injection. All parameters showed no significant difference among the each group, except for the value of bicarbonate ion between the sham group and the anti-HMGB1 mAb-treated group. Values present the means ± SE. **P* < 0.05 compared with the sham group. Results are shown for the sham group (Sham, n = 5), the control IgG-treated group (Cont IgG, n = 5), and the anti-HMGB1 mAb-treated group (α-HMGB1, n = 5). BP: blood pressure [mmHg]; HR: heart rate [bpm]; ICP: intracranial pressure [mmHg]; pH: potential hydrogen; pCO_2: partial pressure of carbon dioxide [mmHg]; pO_2: partial pressure of oxygen [mmHg]; BE: base excess [mmol/L]; HCO_3: bicarbonate ion [mmol/L]; Na: sodium [mmol/L]; K: potassium [mmol/L]; iCa: ionized calcium [mmol/L]; Glu: glucose [mg/dL]; Hct: hematocrit [%]; Hb: hemoglobin [g/dl]; N.D.: not detected.

Supplementary table S2: Primer sequences for quantitative RT-PCR

Gene name	Forward sequence (5' to 3')	Reverse sequence (5' to 3')
α_{1A}-AR	GAATGTCCTGCGAATCCAGT	GATTGGTCCTTTGGCACTGT
AT_1 receptor	ACCAGGTCAAGTGGATTTCG	ATCACCACCAAGCTGTTTCC
eNOS	TGACCCTCACCGATACAACA	CTGGCCTTCTGCTCATTTTC
ET_A receptor	CGTCTTCTGCTTGGTTGTCA	GCAACAGAGGCATGACTGAA
GAPDH	AGACAGCCGCATCTTCTTGT	CTTGCCGTGGGTAGAGTCAT
HMGB1	CCGGATGCTTCTGTCAACTT	TTGATTTTTGGGCGGTACTC
IL-6	CCGGAGAGGAGACTTCACAG	ACAGTGCATCATCGCTGTTC
iNOS	CACCTTGGAGTTCACCCAGT	ACCACTCGTACTTGGGATGC
PAR1	GTTGGATAGTGGGCCGTAGA	TTAGCTGATAGGCCGTGCTT
TLR4	TGCTCAGACATGGCAGTTTC	TCAAGGCTTTTCCATCCAAC
TNF-α	GCCCAGACCCTCACACTC	CACTCCAGCTGCTCCTCT
TXA2 receptor	AGGAGCCTGAATGTTTGGTG	TGAGACAGACGCGGACTATG

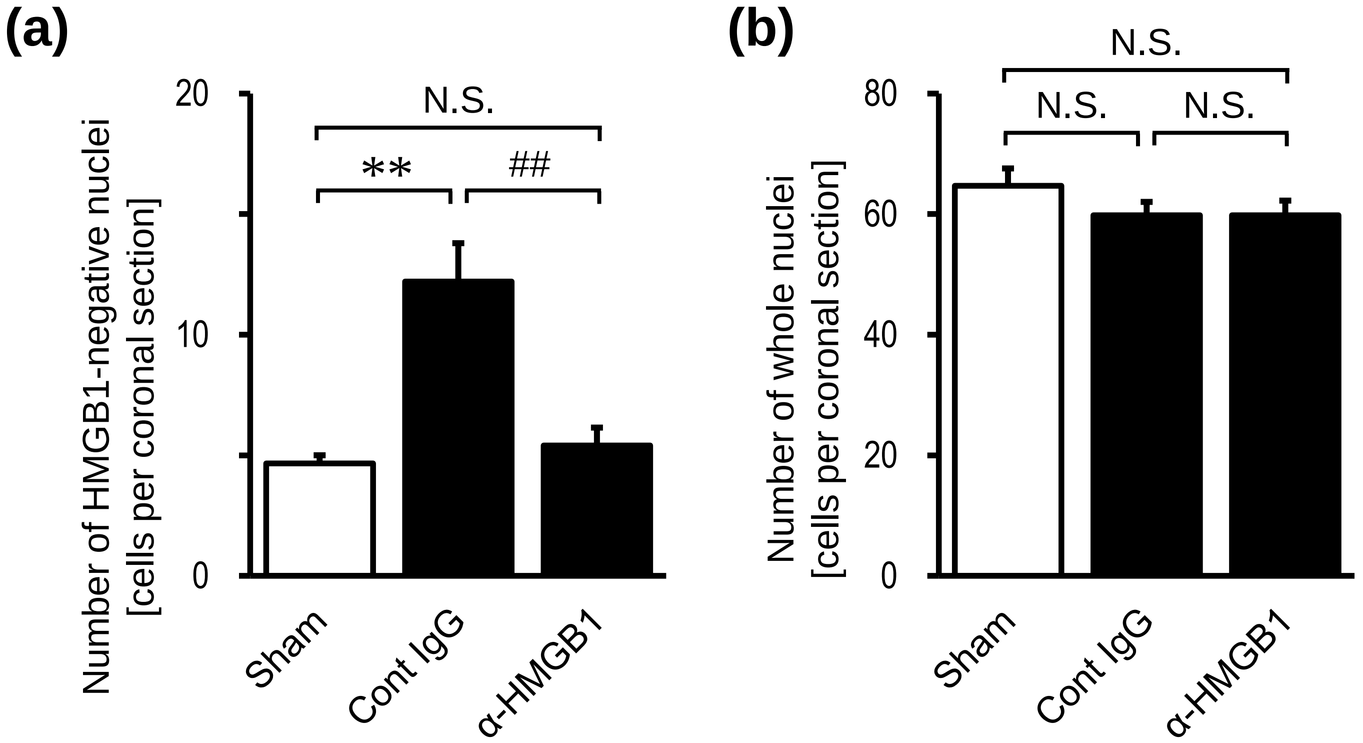

Supplementary figure S1: Effects of anti-HMGB1 mAb on the reduction of nuclear HMGB1 in the BA of SAH rats. Effects of anti-HMGB1 mAb were examined at 48 hr after the SAH in rats treated with anti-HMGB1 mAb or control IgG at both 5 min and 24 hr after blood injection. The whole brain including the BA was prepared as coronal sections at a site 7 mm posterior from the bregma, and subjected to immunostaining for HMGB1 (Alexa Fluor 555) or α-smooth muscle actin (α-SMA) (Alexa Fluor 488) in the BA. All sections were counterstained with DAPI. The extranuclear translocation of HMGB1 was evaluated quantitatively by counting the number of HMGB1-negative nuclei (a) and DAPI-positive nuclei (b) per a whole coronal section of the BA. Results are shown for the sham group (Sham, n = 3), the control IgG-treated group (Cont IgG, n = 5), and the anti-HMGB1 mAb-treated group (α-HMGB1, n = 5). Values represent the means ± SE. $^{**}P < 0.01$ compared with the sham group. $^{\#\#}P < 0.01$ compared with the control IgG-treated group. N.S.: Not significant.

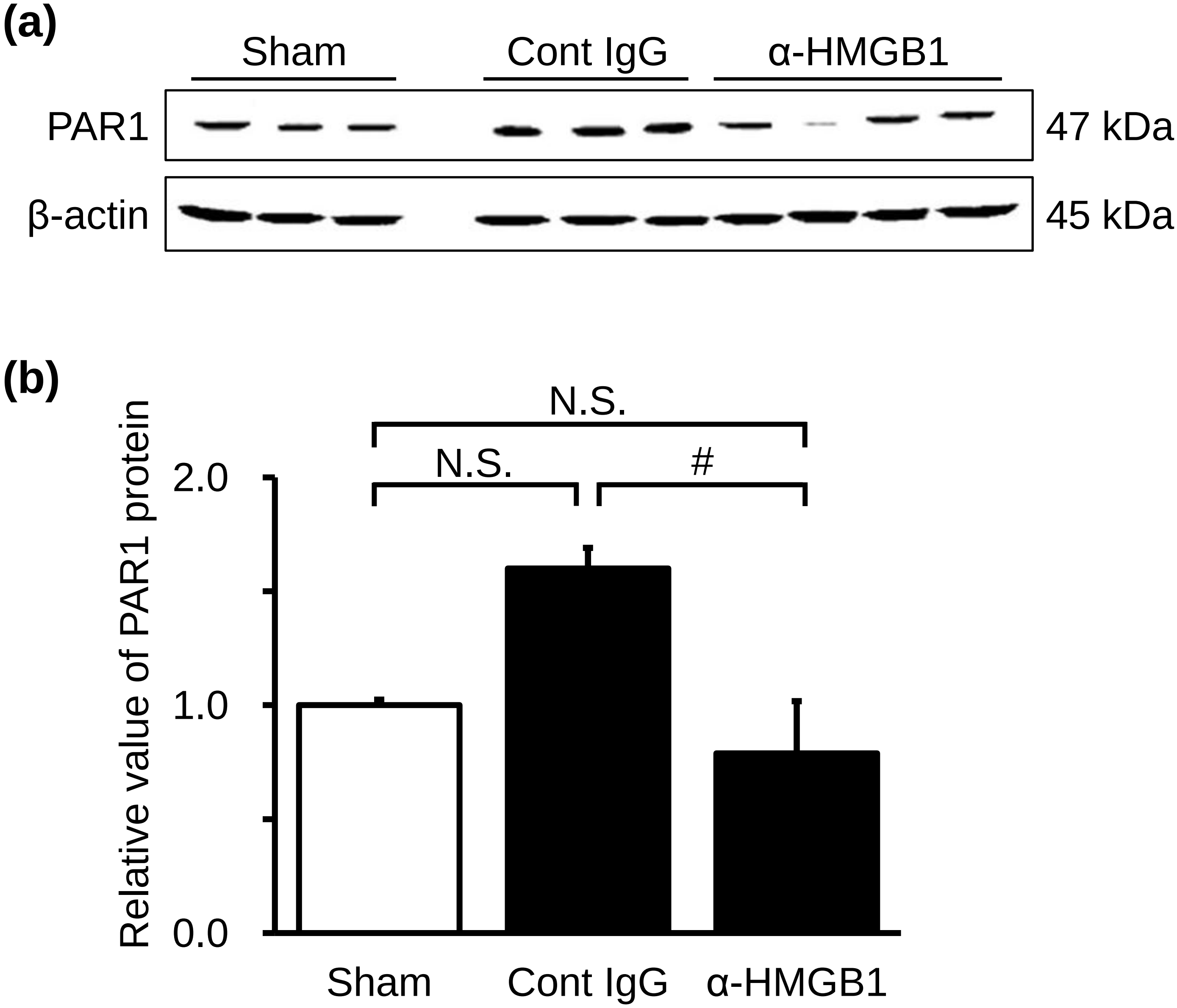

Supplementary figure S2: Effect of anti-HMGB1 mAb on the protein volume of PAR1 in the BA of SAH rats. The BA was isolated at 48 hr after the SAH in rats treated with administration of anti-HMGB1 mAb or control IgG at both 5 min and 24 hr after blood injection. (a) Whole protein was extracted from the isolated BA with RIPA buffer and subjected to immunoblot analysis with anti-thrombin receptor rabbit polyclonal antibody (Abcam, Cambridge, UK) or anti-β-actin rabbit polyclonal antibody (Cell Signaling, Danvers, MA, USA). (b) The level of the changes in the PAR1 protein was normalized by that of β-actin. Results are shown for the sham group (Sham, n = 3), the control IgG-treated group (Cont IgG, n = 3), and the anti-HMGB1 mAb-treated group (α-HMGB1, n = 4). Values represent the means ± SE. $^{\#}P < 0.05$ compared with the control IgG-treated group. N.S.: Not significant.

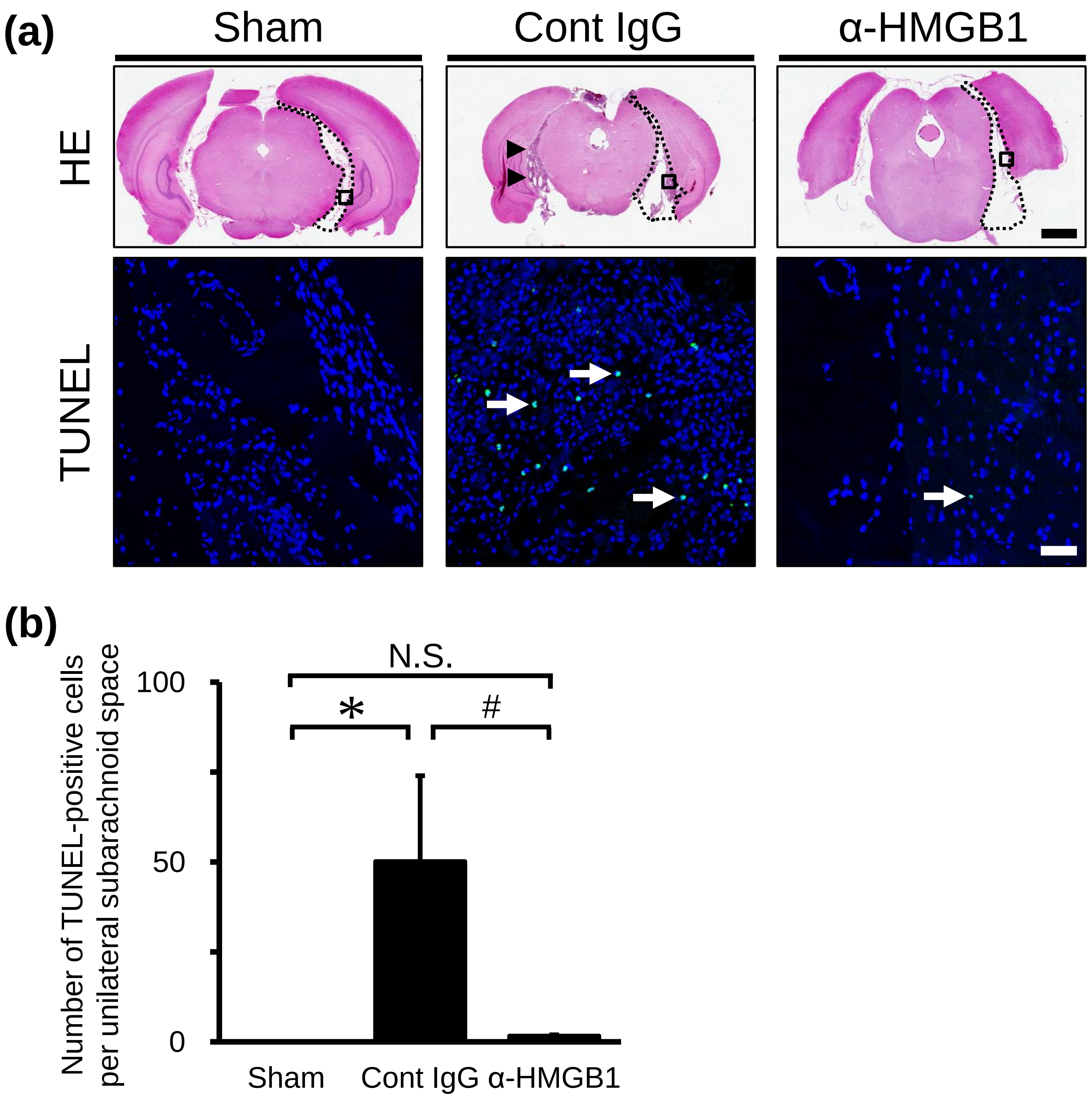
(a)
Sham
Cont IgG
α-HMGB1
HE
TUNEL
(b)
N.S.
*
#
100
50
0
Number of TUNEL-positive cells per unilateral subarachnoid space
Sham
Cont IgG
α-HMGB1

Supplementary figure S3: Effect of anti-HMGB1 mAb on cell death in the brain of SAH rats.

Coronal sections of the whole brain were prepared at 48 hr after the SAH in rats treated with anti-HMGB1 mAb or control IgG at both 5 min and 24 hr after blood injection. A terminal deoxynucleotidyl transferase-mediated dUTP nick end-labeling (TUNEL) assay was performed to determine the distribution of cell death using an *In Situ* Apoptosis Detection Kit (Takara Bio, Siga, Japan) according to the manufacturer's protocol.

Interestingly, remarkably greater infiltration of aggregate cells into the subarachnoid space was observed in the brain of the control IgG-treated group compared with the sham group, and the number of dead cells was also significantly higher in the control IgG-treated group. These cell distributions appeared to correlate with inflammatory brain damage, and in fact they were clearly reduced by the administration of anti-HMGB1 mAb.

(a) Upper panels and lower panels show hematoxylin-eosin (HE) staining and TUNEL assay (FITC) with DAPI staining, respectively. Arrowheads indicate aggregate cells in the subarachnoid space. The dotted area indicates the subarachnoid space. Solid squares indicate the position of the representative TUNEL images shown in the lower panels. Arrows indicate TUNEL-positive cells in the subarachnoid space. (b) Cell death of the aggregate cells was evaluated by counting the TUNEL-positive cells in the unilateral subarachnoid space. The scale bars in black or white indicate 500 μm or 50 μm, respectively. Results are shown for the sham group (Sham, n = 3), the control IgG-treated group (Cont IgG, n = 3), and the anti-HMGB1 mAb-treated group (α-HMGB1, n = 4). Values represent the means ± SE. $^{*}P < 0.05$ compared with the sham group. $^{\#}P < 0.05$ compared with the control IgG-treated group. N.S.: Not significant.

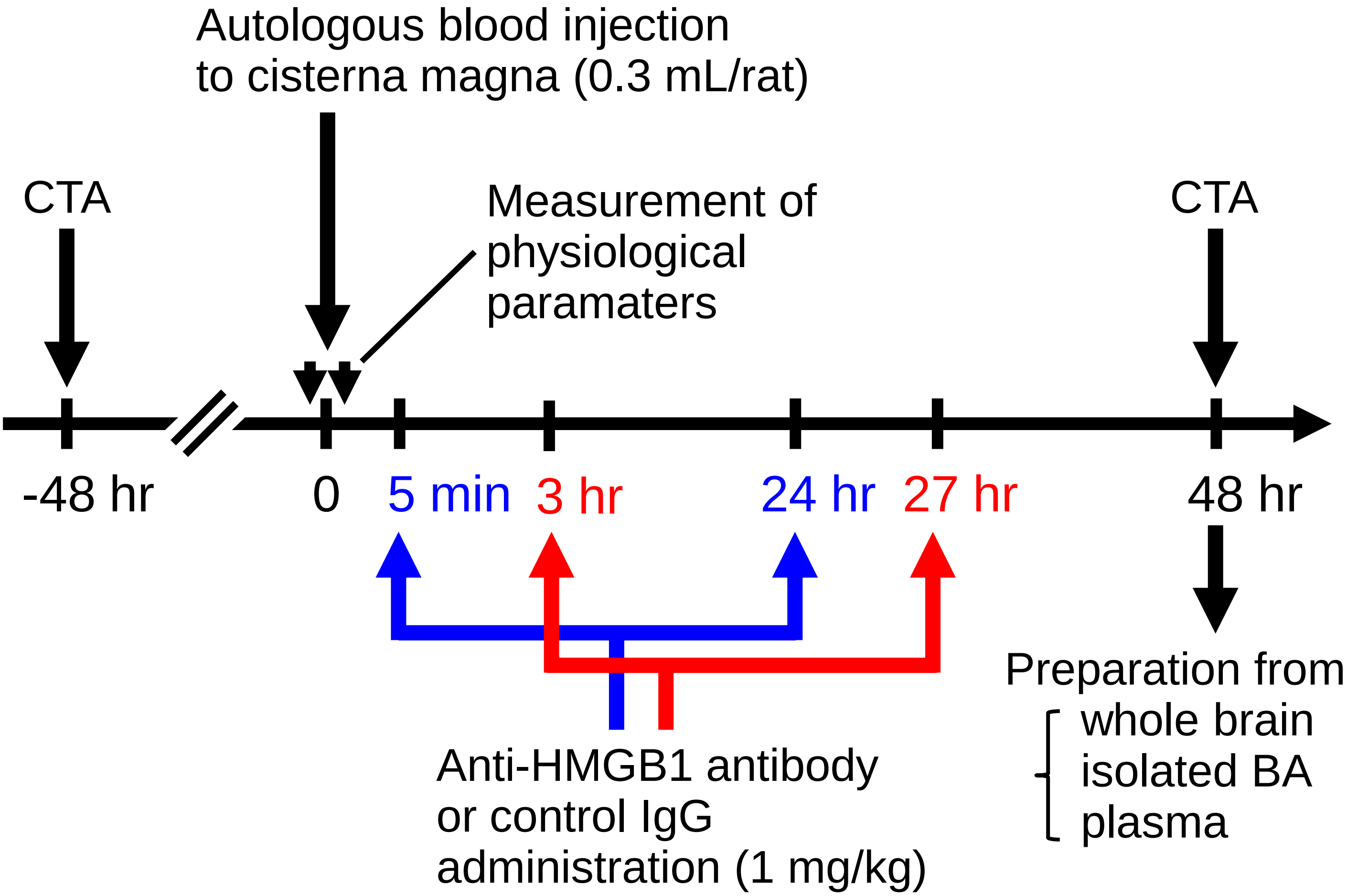

Supplementary figure S4: Schematic outline of the SAH procedures. The time schedule shows the experimental procedures for SAH rats described in the Materials and Methods. The treatment with anti-HMGB1 mAb or control IgG was started at 5 min or 3 hr after the induction of SAH.
